# TNF-α Increases Production of Reactive Oxygen Species through Cdk5 Activation in Nociceptive Neurons

**DOI:** 10.3389/fphys.2018.00065

**Published:** 2018-02-06

**Authors:** Rodrigo Sandoval, Pablo Lazcano, Franco Ferrari, Nicolás Pinto-Pardo, Christian González-Billault, Elías Utreras

**Affiliations:** ^1^Laboratory of Molecular and Cellular Mechanisms of Pain, Department of Biology, Faculty of Science, Universidad de Chile, Santiago, Chile; ^2^Doctorate in Biomedicine, Universidad de los Andes, Santiago, Chile; ^3^Laboratory of Cellular and Neuronal Dynamics, Department of Biology, Faculty of Science, Universidad de Chile, Santiago, Chile; ^4^Center for Geroscience, Brain Health and Metabolism (GERO), Santiago, Chile; ^5^The Buck Institute for Research on Aging, Novato, CA, United States

**Keywords:** Cdk5, NOX, TNF-α, pain signaling, ROS

## Abstract

The participation of reactive oxygen species (ROS) generated by NOX1 and NOX2/NADPH oxidase has been documented during inflammatory pain. However, the molecular mechanism involved in their activation is not fully understood. We reported earlier a key role of Cyclin-dependent kinase 5 (Cdk5) during inflammatory pain. In particular, we demonstrated that TNF-α increased p35 expression, a Cdk5 activator, causing Cdk5-mediated TRPV1 phosphorylation followed by an increment in Ca^2+^ influx in nociceptive neurons and increased pain sensation. Here we evaluated if Cdk5 activation mediated by p35 transfection in HEK293 cells or by TNF-α treatment in primary culture of nociceptive neurons could increase ROS production. By immunofluorescence we detected the expression of catalytic subunit (Nox1 and Nox2) and their cytosolic regulators (NOXO1 and p47^phox^) of NOX1 and NOX2/NADPH oxidase complexes, and their co-localization with Cdk5/p35 in HEK293 cells and in nociceptive neurons. By using a hydrogen peroxide sensor, we detected a significant increase of ROS production in p35 transfected HEK293 cells as compared with control cells. This effect was significantly blocked by VAS2870 (NADPH oxidase inhibitor) or by roscovitine (Cdk5 activity inhibitor). Also by using another ROS probe named DCFH-DA, we found a significant increase of ROS production in nociceptive neurons treated with TNF-α and this effect was also blocked by VAS2870 or by roscovitine treatment. Interestingly, TNF-α increased immunodetection of p35 protein and NOX1 and NOX2/NADPH oxidase complexes in primary culture of trigeminal ganglia neurons. Finally, the cytosolic regulator NOXO1 was significantly translocated to plasma membrane after TNF-α treatment and roscovitine blocked this effect. Altogether these results suggest that Cdk5 activation is implicated in the ROS production by NOX1 and NOX2/NADPH oxidase complexes during inflammatory pain.

## Introduction

During tissue damage, several inflammatory mediators as tumor necrosis factor-α (TNF-α) are locally released, activating signaling pathways in sensory neurons that increase peripheral sensitization and pain signaling (Khan et al., 2008; Sessle, 2011; Rozas et al., 2016). Activated protein kinases such as PKC, PKA, and Cdk5 are involved in peripheral sensitization through phosphorylation of several ion channels expressed on nociceptive neurons (Basbaum et al., 2009; Rozas et al., 2016; Coddou et al., 2017). Cdk5 is an essential kinase in brain development and function (Dhariwala and Rajadhyaksha, 2008; Utreras et al., 2009a; Contreras-Vallejos et al., 2012). Interestingly, our group reported earlier that Cdk5 plays a crucial role during inflammatory pain signaling (Utreras et al., 2009a, 2011; Prochazkova et al., 2013; Rozas et al., 2016; Coddou et al., 2017). Cdk5 is a proline-directed serine/threonine kinase, mostly active in post-mitotic neurons, where its specific activators p35 and p39 are mainly expressed (Lew et al., 1994; Dhariwala and Rajadhyaksha, 2008; Utreras et al., 2009c). We reported also that cytokines such as TNF-α or TGF-β1 up-regulate p35 expression and Cdk5 kinase activity with a subsequent phosphorylation of transient receptor potential vaniloid 1 (TRPV1) and purinergic P2X2a receptor (P2X2aR), which are important receptors channels involved during inflammatory pain signaling (Utreras et al., 2009b, 2011, 2012, 2013; Prochazkova et al., 2013; Rozas et al., 2016; Coddou et al., 2017).

On the other hand, reactive oxygen species (ROS) represent important players during inflammation (Mittal et al., 2014). There is growing evidence supporting ROS as molecules that contribute to pain hypersensitivity (Kallenborn-Gerhardt et al., 2013). Interestingly, antioxidant therapy has been used to overcome painful effects developed in inflammatory pain models (Khattab, 2006; Lauro et al., 2016; Wu et al., 2017). There are several enzymatic systems that generate ROS such as lipoxygenases, xanthine oxidases, cyclooxygenases, cytochrome P450 monooxygenases, nitric oxide synthases, and NADPH oxidases (NOX) (Holmström and Finkel, 2014; Bórquez et al., 2016; Wilson et al., 2017). NOX enzymes belong to the NOX family that is composed by seven members (NOX1-5 and Duox 1-2). Interestingly, Nox1, Nox2, and Nox4 catalytic enzymes have been associated with pain signaling (Kallenborn-Gerhardt et al., 2012, 2013, 2014; Geis et al., 2017). NOX enzymes reside at the plasma membrane in tight association with the integral membrane protein p22^phox^. When NOX complex becomes active, it generates ROS by a catalytic transfer of electrons from NADPH to O_2_ to form superoxide anion and hydrogen peroxide (Sumimoto et al., 2005). Nox1 activation occurs by PKC-mediated phosphorylation of the cytosolic subunit Nox organizer 1 (NOXO1) allowing NOXO1-p22^phox^ interaction (Debbabi et al., 2013). Similarly, activation of Nox2 (Gp91^phox^) needs the recruitment of the regulatory cytosolic subunits p47^phox^, p67^phox^, and Rac1 (El-Benna et al., 2008). Multiple serine phosphorylation of p47^phox^ by PKC is a key step that induces association with p22^phox^ at the plasma membrane and activation of the complex (Fontayne et al., 2002; Meijles et al., 2014). In the present work we describe a new role of Cdk5 in the modulation of redox balance in nociceptive neurons. Thus, activation of Cdk5 in HEK293 cells and primary sensory neurons was related to increased ROS production. Interestingly, this effect was blocked by inhibition of NOX complex or Cdk5 kinase activity, suggesting a molecular link between Cdk5 and NOX-mediated ROS production. Implications of these findings could address additional roles of Cdk5 in pain signaling.

## Materials and methods

### Transfection of HEK293 cells

HEK293 cells (ATCC#CRL-1573) were grown in Dulbecco Modified Eagle Medium (DMEM) containing 10% of fetal bovine serum (FBS) and penicillin/streptomycin (100 mg/mL) (Invitrogen, Carlsbad CA). HEK293 cells were transiently co-transfected with mouse CMV-p35 (Coddou et al., 2017) and HyPer constructs (Evrogen, Moscow, Russia) by using Lipofectamine 2000 reagent (Invitrogen, Carlsbad CA) and treated with roscovitine (30 μM, Sigma-Aldrich, Saint Louis, MO) or VAS2870 (1 μM, Calbiochem, CA USA) during 24 h and 1 h, respectively.

### Primary culture of mouse nociceptive neurons

Nociceptive neurons were cultured as described previously (Coddou et al., 2017). Briefly, trigeminal ganglia (TG) and dorsal root ganglia (DRG) were dissected out from 7 to 10 month-old C57/FVB mice and incubated with collagenase XI (0.66 mg/mL) and dispase II (3 mg/mL, Sigma-Aldrich, Saint Louis, MO) in an INCmix solution (NaCl 155 mM; K_2_HPO_4_ 1.5 mM; HEPES 10 mM; glucose 5 mM; at pH 7.4). Enzymatic digestion was performed for 45 min at 37°C in 5% CO_2_, and consecutively treated with DNase I (100 μg/mL, Roche Diagnostic, Indianapolis, IN) during 10 min at 37°C. TG/DRG cell suspensions were separated over discontinuous 28–12.5% Percoll gradients (GE Healthcare). Isolated cells were cultured in minimum essential media (MEM) supplemented with 10% FBS, penicillin/streptomycin (100 mg/mL), and MEM-Vit (Invitrogen, Carlsbad CA). Cells were plated on 12-mm poly-L-lysine-coated glass coverslips and cultured for 2 days *in vitro* (2DIV). To evaluate the involvement of Cdk5 activation by TNF-α and NOX signaling, TG and DRG primary cultures (2DIV) were treated with TNF-α (25–50 ng/mL, Sigma-Aldrich, Saint Louis, MO) in the presence or absence of roscovitine (20 μM) or VAS2870 (1 μM) during 24 h and 1 h, respectively. Animal experiments were conducted in accordance with the principles and procedures of the Ethics Committee of the Biology Department, Faculty of Sciences, Universidad de Chile, Santiago, Chile.

### Immunofluorescence assays

HEK293 cells transfected with p35 or primary cultures of TG and DRG neurons were washed with warm PBS for 5 min and fixed with a 4% PFA-4% sucrose solution in PBS at 37°C for 20 min. Cells were washed and permeabilized for 5 min with 0.2% Triton X-100-PBS solution. After washout with PBS, cells were blocked with a 5% BSA solution in PBS at room temperature for 1 h. Primary antibodies were used at following concentrations: anti-Cdk5 mouse DC17 (1:100), anti-p35 rabbit C19 (1:100), anti-Nox1 goat sc-292094 (1:100), anti-NOXO1 rabbit sc-5821 (1:100), anti-NOXA1 rabbit sc-160597-R (1:100), anti-p47^phox^ rabbit sc-14015 (1:100), anti-MAP1B goat N-19 (1:200), anti-p35 goat A-18 (1:100) (from Santa Cruz Biotechnology); anti-gp91^phox^ mouse ab109366 (1:100), and anti-p22^phox^ rabbit ab75941 (1:100) (from Abcam); anti-βIII tubulin mouse clone G7121 (1:1000) (from Promega); p35 rabbit C64B10 (1:100) (from Cell Signaling Technology, Denver, USA). All primary antibodies were diluted in 1% BSA solution and incubated overnight at 4°C. The coverslips were washed with PBS and then incubated with corresponding Alexa Fluor-conjugated secondary antibodies. We used the following secondary antibodies:-Donkey anti-Mouse IgG (H+L) Highly Cross-Adsorbed Secondary Antibody, Alexa Fluor 488 #A21202-Donkey anti-Rabbit IgG (H+L) Highly Cross-Adsorbed Secondary Antibody, Alexa Fluor 488 #A21206-Donkey anti-Rabbit IgG (H+L) Highly Cross-Adsorbed Secondary Antibody, Alexa Fluor 546 #A10040-Donkey anti-Mouse IgG (H+L) Highly Cross-Adsorbed Secondary Antibody, Alexa Fluor 546 #A10036-Donkey anti-Goat IgG (H+L) Cross-Adsorbed Secondary Antibody, Alexa Fluor 633 #A21082 (Molecular Probes, Life Technologies, Grand Island, NY) in combination with Dapi (Thermo Fisher Scientific) for 1 h at room temperature. Finally, coverslips were washed with PBS and mounted on a slide with FluorSave (Calbiochem). Images were acquired using confocal microscopy (LSM 710 Meta Model, Carl Zeiss Microscopy) and processed with the LSM Image Browser (Carl Zeiss Microscopy) software.

### Immunofluorescence quantification analysis

As an estimation of protein amount in individual neurons, the fluorescence intensity was quantified as reported earlier (McCloy et al., 2014). Confocal images (40X) acquired from immunofluorescences were processed by using ImageJ 1.46r software (NIH, Bethesda, MD) and individual ROIs were assigned to each neuron and integrated density was obtained for each fluorescent emission.

### Hydrogen peroxide measurement in transfected HEK293 cells

We evaluated hydrogen peroxide content in HEK293 cells by using HyPer sensor as previously reported (Belousov et al., 2006). HEK293 cells co-transfected with HyPer and CMV-p35 plasmids during 24 h were fixed in 4% paraformaldehyde/4% sucrose solution in PBS. Then, cells were excited at 488 and 405 nm and emission was collected at 505–530 nm in a confocal microscopy (LSM 710 Meta Model, Carl Zeiss Microscopy). Fluorescence emission from excitation at 488 nm was divided by fluorescence emission at 405 nm excitation (488:405) as a measure of the hydrogen peroxide content (Belousov et al., 2006).

### Neuronal ROS measurement

To evaluate intracellular ROS levels, primary cultures of mouse TG and DRG neurons were incubated simultaneously with CellTracker^TM^ Orange dye (CMTMR 1 μM, Thermo Fisher Scientific) and 2′,7′-Dichlorofluorescin diacetate (DCFH-DA 1 μM, Sigma) for 20 min at 37°C similarly to previously reported (Wilson et al., 2015). DCFH-DA detects intracellular oxidative species by increasing fluorescence emission after oxidation (Lebel and Bondy, 1990). Sensory neurons were fixed and mounted to measure the fluorescence by excitation at 488 nm and emission acquisition at 505–530 nm. Fluorescence from CMTMR dye incorporated in neurons was used to normalize DCFH-DA emission, and was acquired by excitation at 543 nm and emission acquisition at 548–679 nm.

### Translocation to plasma membrane analysis

To estimate plasma membrane translocation of NOX cytosolic subunits (NOXO1 and p47^phox^), we drew a line that crosses the 2D surface of each neuron from end to end (**Figure 6A**). Gray value was obtained along the drawn line to estimate the amount of protein in zones near and far of plasma membrane (peripheral and centric areas, respectively). Mean gray value of centric areas were calculated as the mean of all gray values comprising the medial portion of the drawn line (30% of total line). Peripheral areas were defined as the first 4 μm from the periphery to the center in the drawn line. Mean gray value was calculated in this short section alike centric areas. Finally, the mean gray value of peripheral area (two per neuron) was normalized to corresponding centric area mean gray value (**Figure 6A**).

### Western blot analysis

Protein extracts from HEK293 cells transfected with p35 were obtained in T-PER buffer (Pierce, Rockford, IL) with Complete Mini protease inhibitor cocktail tablets and PhosSTOP phosphatase inhibitor cocktail tablets (Roche Diagnostic, Indianapolis, IN). Protein extracts were resolved in 12% SDS-PAGE gels and transferred to nitrocellulose membranes (Invitrogen, Carlsbad, CA). Membranes were soaked in blocking buffer (5% non-fat dry milk in Tris-Buffered Saline (TBS) with 0.05% Tween-20 (TBS-T)) for 1 h at room temperature, and then incubated overnight at 4°C, with primary antibody diluted in 1% non-fat dry milk blocking buffer. The membranes were washed in TBS-T and incubated for 1 h at room temperature with the secondary antibodies diluted in 1% non-fat dry milk blocking buffer. Immunoreactivity was detected by using Super-Signal West Pico Chemiluminescent Substrate (Thermo Scientific, Rockford, IL). Western blots were performed by using anti-p35 rabbit C19 (1:250) and anti-Cdk5 mouse DC17 (1:500) from Santa Cruz Biotechniology; anti-α-tubulin mouse (1:10.000) from Sigma. We used secondary antibodies anti-mouse and anti-rabbit coupled to Horseradish Peroxidase from Santa Cruz Biothecnology. The optical densities of the bands were quantified using an image analysis system with ImageJ 1.46r software (NIH, Bethesda, MD).

### Statistical analysis

All experiments were performed a minimum of three times. All graphs show the mean ± *SD*. Statistical evaluation was performed with GraphPad Prism software, version 6.1 (GraphPad, San Diego, CA). Significant differences between experiments were assessed by an unpaired *t*-test or a one-way analysis of variance with a Bonferroni's multiple comparison test, where α was set to 0.05.

## Results

### Endogenous expression of NOX complexes in HEK293 cells

**Figure 1:**
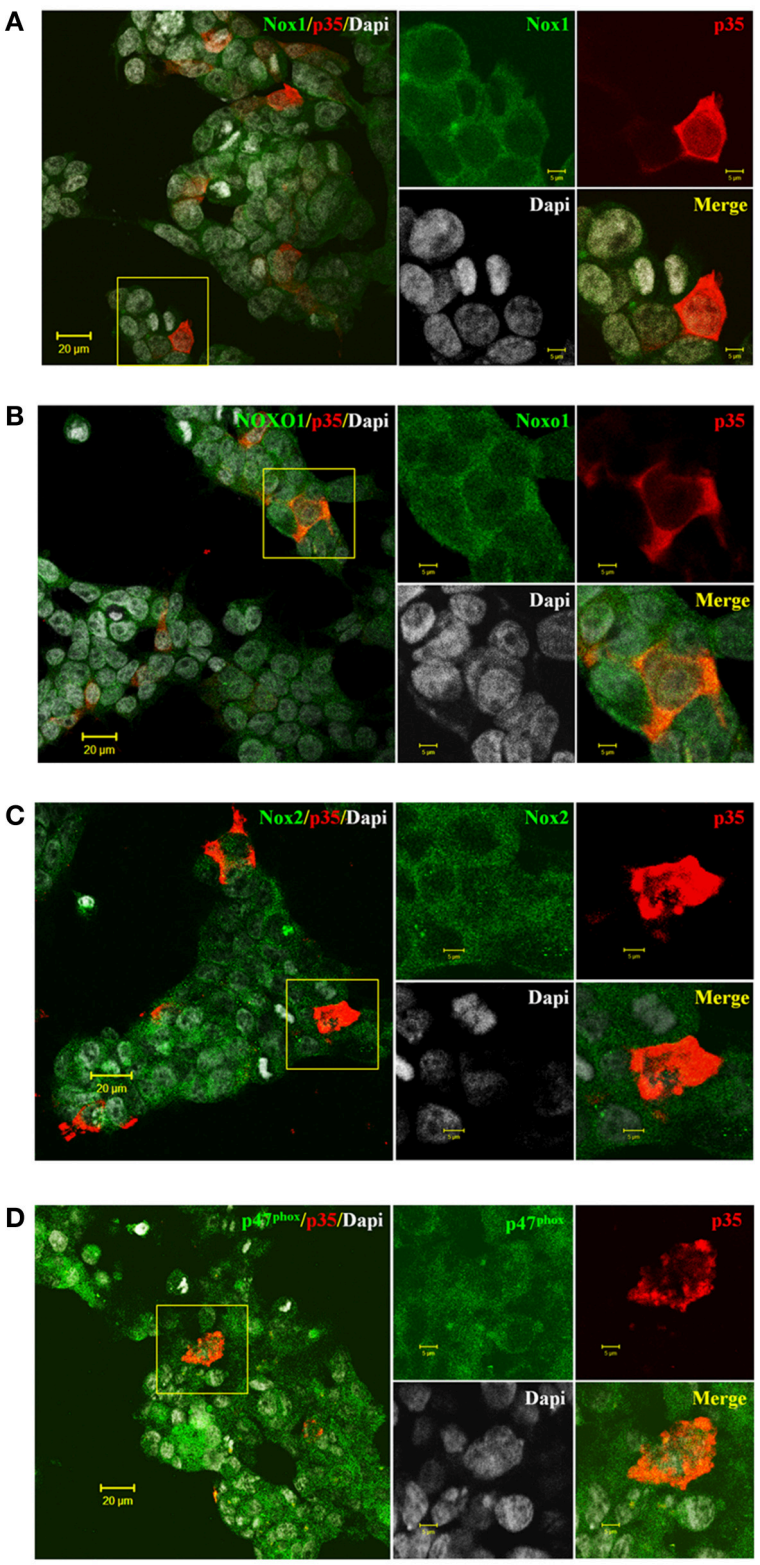
Endogenous expression of Nox1/NOXO1 and Nox2/p47^phox^ in HEK293 cells transfected with p35 plasmid**. (A,C)** Representative immunofluorescences showing catalytic subunits Nox1 and Nox2 (in green, respectively) co-localizing with p35 (in red) in transfected HEK293 cells. **(B,D)** Representative immunofluorescences of regulatory subunits NOXO1 and p47^phox^ (in green, respectively) co-localizing with p35 (in red) in transfected HEK293 cells. Inset for each figure showing staining by separate in larger magnification. DAPI for nuclear staining (gray). Scale bars are 20 and 5 μm insets.

**Figure 2:**
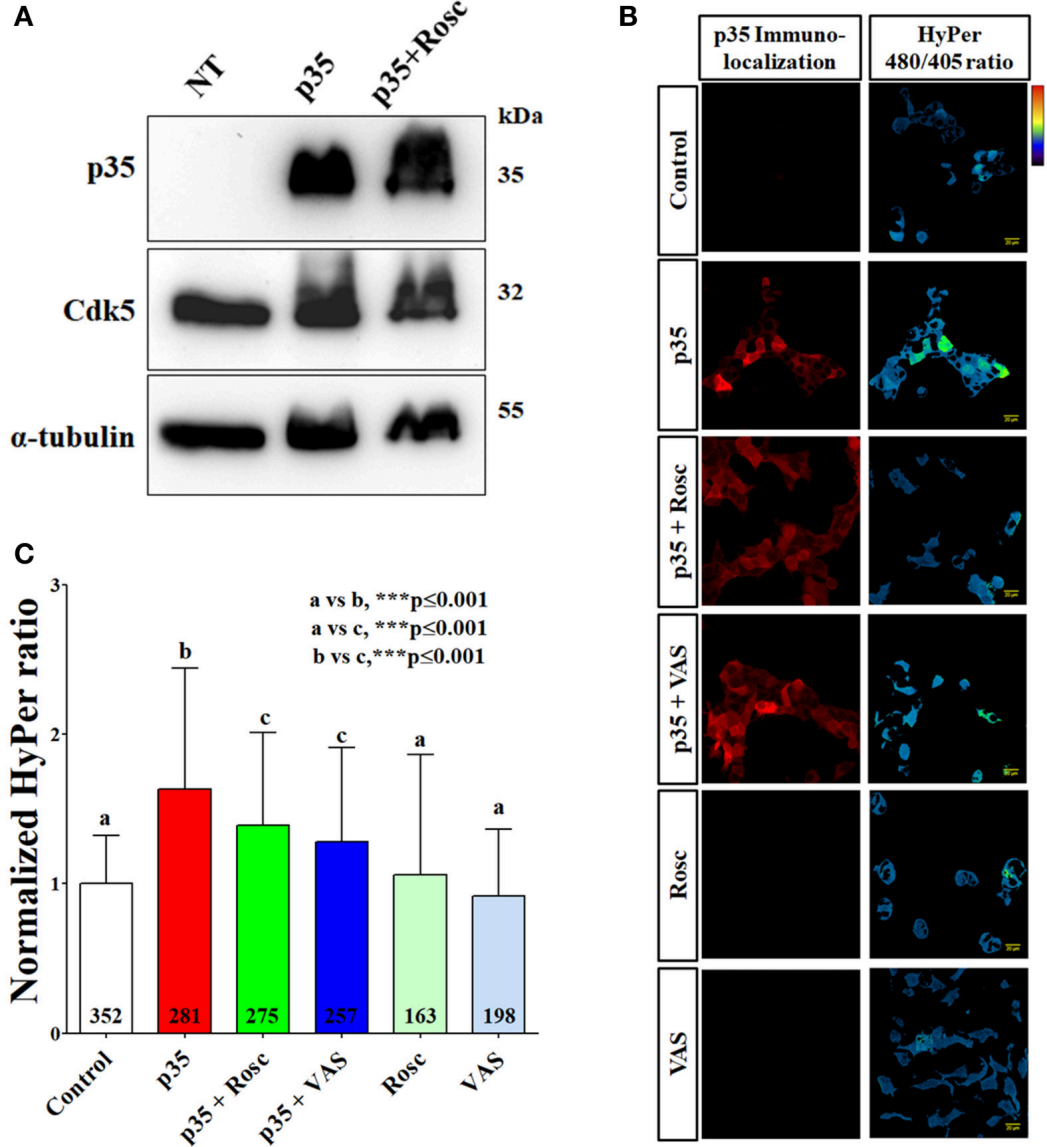
Increased Cdk5 kinase activity in HEK293 cells transfected with p35 produced elevated hydrogen peroxide content measured by HyPer probe. **(A)** Western blot analysis against p35, Cdk5, and α-tubulin from HEK293 cells untransfected (UT), transfected with p35 (p35), and transfected with p35 and treated with roscovitine (30 μM) (p35 + Rosc) during 24 h. **(B)** Representative immunofluorescences of p35 (left) and HyPer 480/405 ratio (right) from HEK293 cells transfected with p35 and HyPer, treated with roscovitine or VAS2870. Thermal scale: increased hydrogen peroxide content toward red. **(C)** Normalized HyPer 480/405 ratio in same conditions of **(B)**. Scale bars are 20 μm. The bar graphs represent mean ± SD of *n* = 4–7 different cell cultures. Number of cells analyzed is showed inside each column. Statistical differences correspond to a one-way ANOVA with a Bonferroni's multiple comparison test.

To establish Cdk5 participation in NOX1 and NOX2-dependent ROS production, we analyzed the immunolocalization of NOX1 and NOX2/NADPH oxidase complexes in HEK293 cells transfected with p35 that endogenously express Cdk5 (Figure 1). By immunofluorescence we detected co-localization of catalytic (Nox1 and Nox2) and cytosolic subunits (NOXO1 and p47^phox^) with p35 protein. We observed endogenous expression of Nox1, Nox2, NOXO1 and p47^phox^ in plasma membrane and in cytoplasmic regions of HEK293 cells (Figures 1A–D, green label). We also found that HEK293 cells expressed other members of the catalytic NOX core such as p22^phox^ and NOXA1 (data not shown). Most important, we immunodetected p35 in some transfected cells co-localizing with NOX1 and NOX2 complexes (Figures 1A–D, red label). Additionally, we detected endogenous Cdk5 and transfected p35 proteins by Western blot from HEK293 cells in the presence or absence of roscovitine (Figure 2A). We also confirmed that HEK293 cells transfected with p35 have increased Cdk5 kinase activity (data not shown), similarly as previously reported (Zheng et al., 2002).

### Increased cdk5-dependent ROS production in HEK293 cells

To evaluate if ROS production is affected by increased Cdk5 activity in transfected HEK293 cells, we used the genetically encoded biosensor HyPer which detects intracellular production of hydrogen peroxide (Lukyanov and Belousov, 2014; Wilson et al., 2015). Firstly, by immunofluorescence we determined p35 expression in HEK293 cells co-transfected with p35 and HyPer plasmids (Figure 2B). Then, we generated a HyPer map (480/405 nm ratio) for each treatment condition of HEK293 cells. In HEK293 cells overexpressing p35 we found a significant increase of hydrogen peroxide content and this effect was significantly reverted by roscovitine (Figures 2B,C). Interestingly, Cdk5-dependent ROS production was also reverted by NOX inhibitor, VAS2870 (Figures 2B,C), suggesting that NOX complexes represent a source for the elevated ROS observed in this model. The basal hydrogen peroxide production was not affected by roscovitine treatment and only slightly reduced by VAS2870 treatment (Figures 2B,C). These results suggest that production of ROS is dependent, in part, on Cdk5 activity in HEK293 cells expressing active NOX1 and NOX2 complexes.

### Endogenous expression of NOX1 and NOX2 complexes and Cdk5/p35 in primary culture of TG neurons

**Figure 3:**
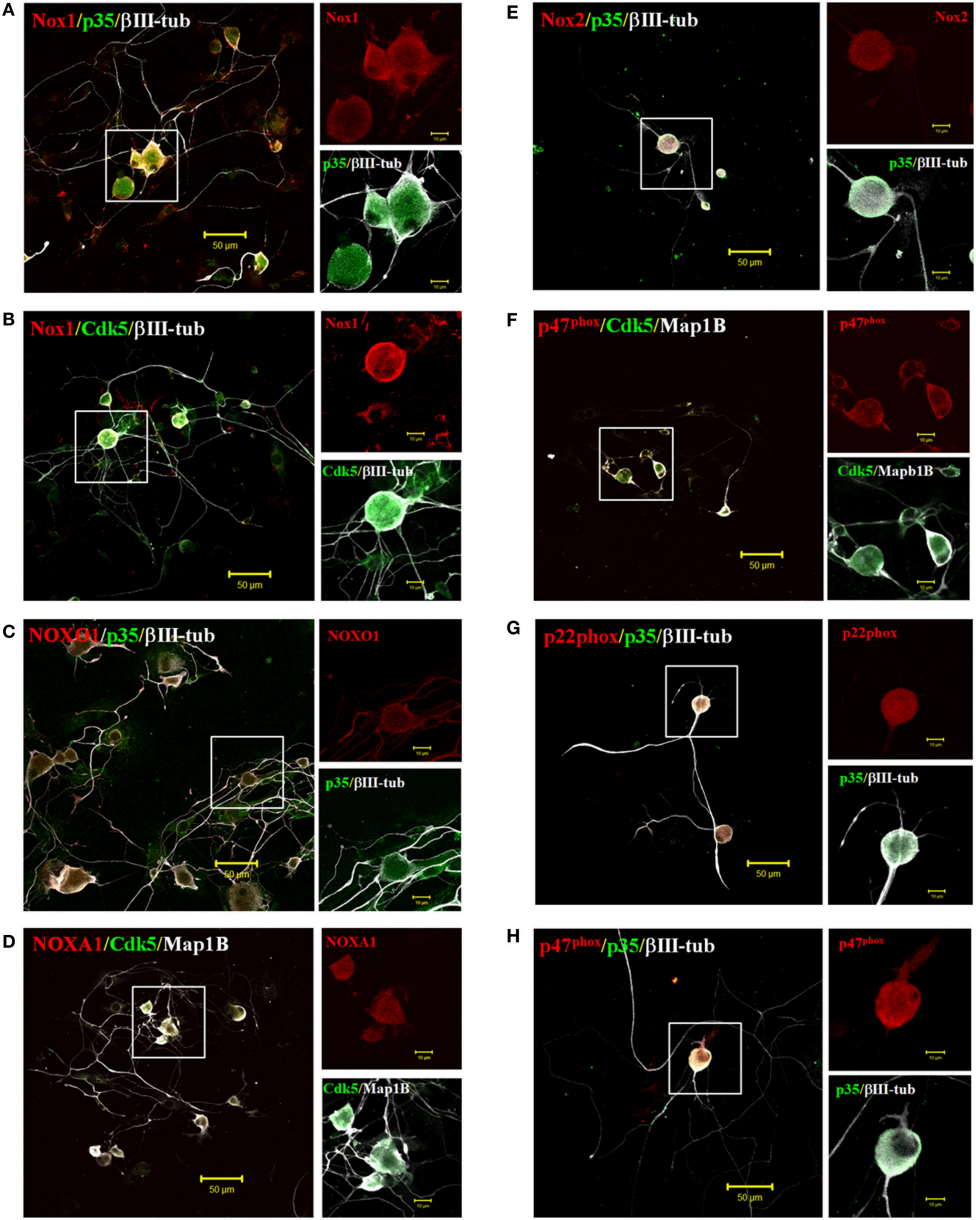
Endogenous expression of NOX1 and NOX2 complexes with Cdk5/p35 in primary culture of trigeminal ganglia neurons. Representative immunofluorescences of catalytic subunit Nox1 **(A,B)** and its regulatory subunit NOXO1 **(C)** and NOXA1 **(D)** in red, co-localizing with Cdk5 and p35 (in green) expressed endogenously in primary culture of trigeminal ganglia neurons. Representative immunofluorescences of catalytic subunit Nox2 **(E)** and its regulatory subunits p47^phox^ **(F,H)** and p22^phox^ **(G)** in red, co-localizing with Cdk5 and p35 (in green) expressed endogenously in primary culture of trigeminal ganglia neurons. βIII-tubulin or MAP1B is a marker of neurons (gray). Scale bars are 50 or 10 μm (insets).

Our previous data suggests that Cdk5 is involved in ROS production mediated by the NOX1 and NOX2 complex in HEK293 cells. Importantly, the production of ROS (Kallenborn-Gerhardt et al., 2013) and also Cdk5 kinase (Utreras et al., 2009a) are active participants during pain signaling. Therefore, we evaluated the co-distribution of Cdk5/p35 with members of NOX1 and NOX2 complexes in sensory neurons. The NOX family members NOX1 and NOX2 have been associated with pain processes (Kallenborn-Gerhardt et al., 2013), consequently we studied their distribution in primary culture of mouse TG neurons. By immunofluorescence, we identified that NOX members of both complexes are highly expressed in neurons of all sizes (small, medium, and large neurons), respect to non-neuronal cells (Figure 3, negative cells for βIII-tubulin). Catalytic (Nox1 and Nox2) and cytoplasmic (NOXO1 and p47^phox^) subunits were mostly found in somata and in neurites, with the exception of NOXO1, which was equally identified in both neuronal regions including large processes (Figures 3A–H). We also observed endogenous Cdk5 and p35 expression in TG neurons and others cells co-localizing with both NOX1/NOX2 complexes (Figures 3A–H), suggesting that Cdk5-dependent ROS production is likely to occur in trigeminal ganglia neurons.

### TNF-α increased ROS production in primary culture of TG and DRG neurons

**Figure 4:**
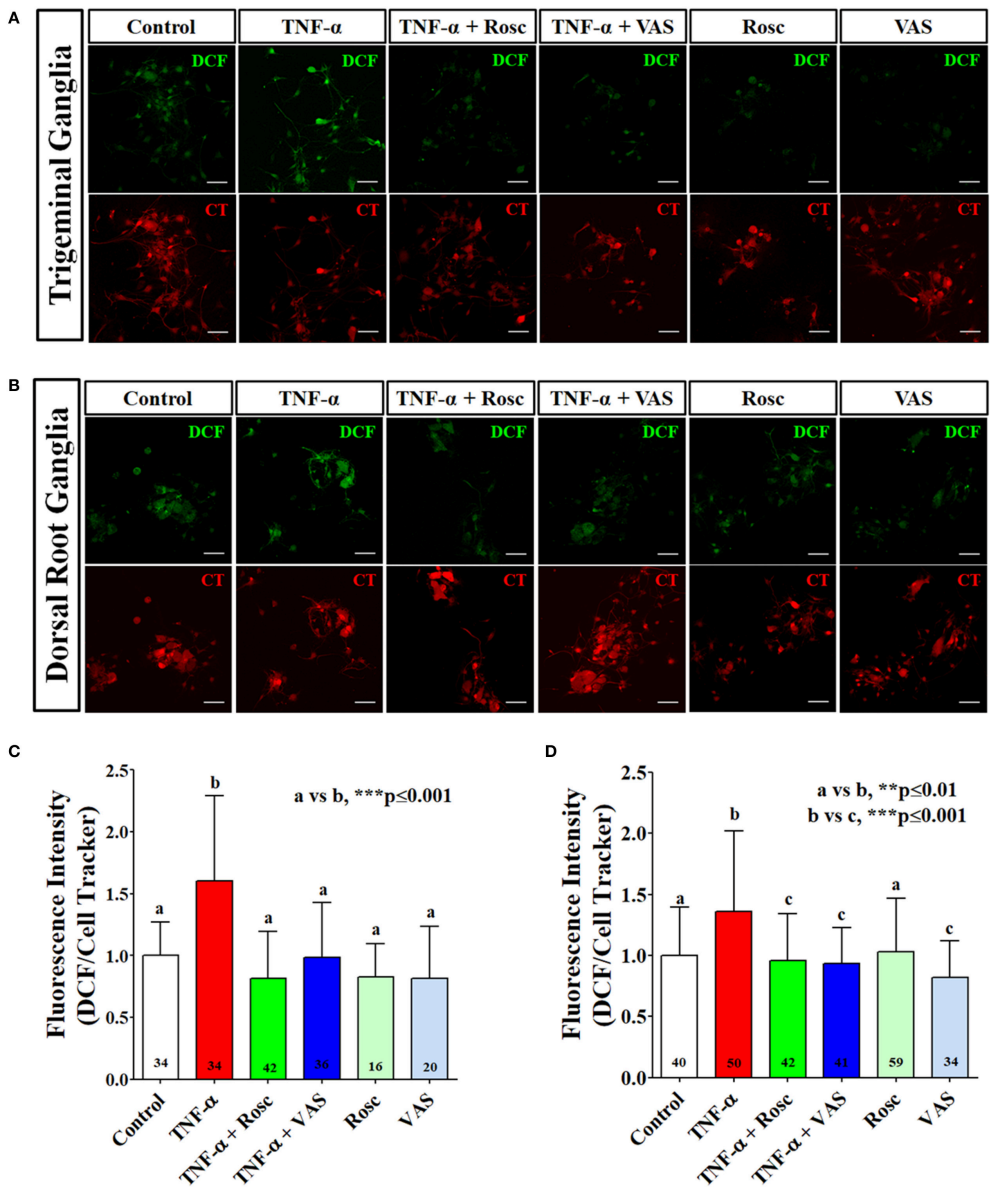
TNF-α increases ROS production in primary culture of mouse TG and DRG neurons. Representative images of primary cultures loaded with DCFH-DA (DCF) and CMTMR (CT) probes from TG **(A)** and DRG **(B)** neurons treated with TNF-α alone or with roscovitine or VAS2870. **(C,D)** Quantification of fluorescence intensity of DCF normalized by CT under each corresponding treatment in TG neurons **(C)** or DRG neurons **(D)**. Scale bars are 20 μm. The bar graphs represent mean ± *SD* of *n* = 3 different experiments. Statistical differences correspond to a one-way ANOVA with a a Bonferroni's multiple comparison test. Number of analyzed neurons for each treatment is inside the corresponding bar.

Previously, we reported that TNF-α increases Cdk5 kinase activity by transcriptional up-regulation of p35 in cell lines and in TG and DRG neurons (Utreras et al., 2009b; Rozas et al., 2016). To evaluate the association between TNF-α-mediated Cdk5 activation and NOX-dependent ROS production in nociceptive neurons, we performed primary cultures of mouse TG and DRG neurons treated with TNF-α and we further measured ROS production by using DCFH-DA probe (Lebel and Bondy, 1990; Wilson et al., 2015) (Figures 4A,B). We found that TNF-α treatment significantly increased the intracellular ROS content. In contrast, this effect was completely blocked by roscovitine, suggesting the involvement of Cdk5 in ROS production in sensory neurons (Figures 4A–D). Interestingly, we found that the source of ROS content in TG and DRG primary culture was NOX-dependent, since VAS2870 also blocked the TNF-α-mediated ROS production (Figures 4A–D). Basal ROS production was not affected by roscovitine treatment and only slightly reduced by VAS2870 in DRG neurons. These data are in agreement with our previous results obtained in HEK293 cells, suggesting that Cdk5 contributes to ROS balance in nociceptive neurons. However, the molecular mechanism involving Cdk5 activity in ROS production through NOX complexes is not clear.

### TNF-α increased expression of NOX1 and NOX2 members in primary culture of mouse TG neurons

**Figure 5:**
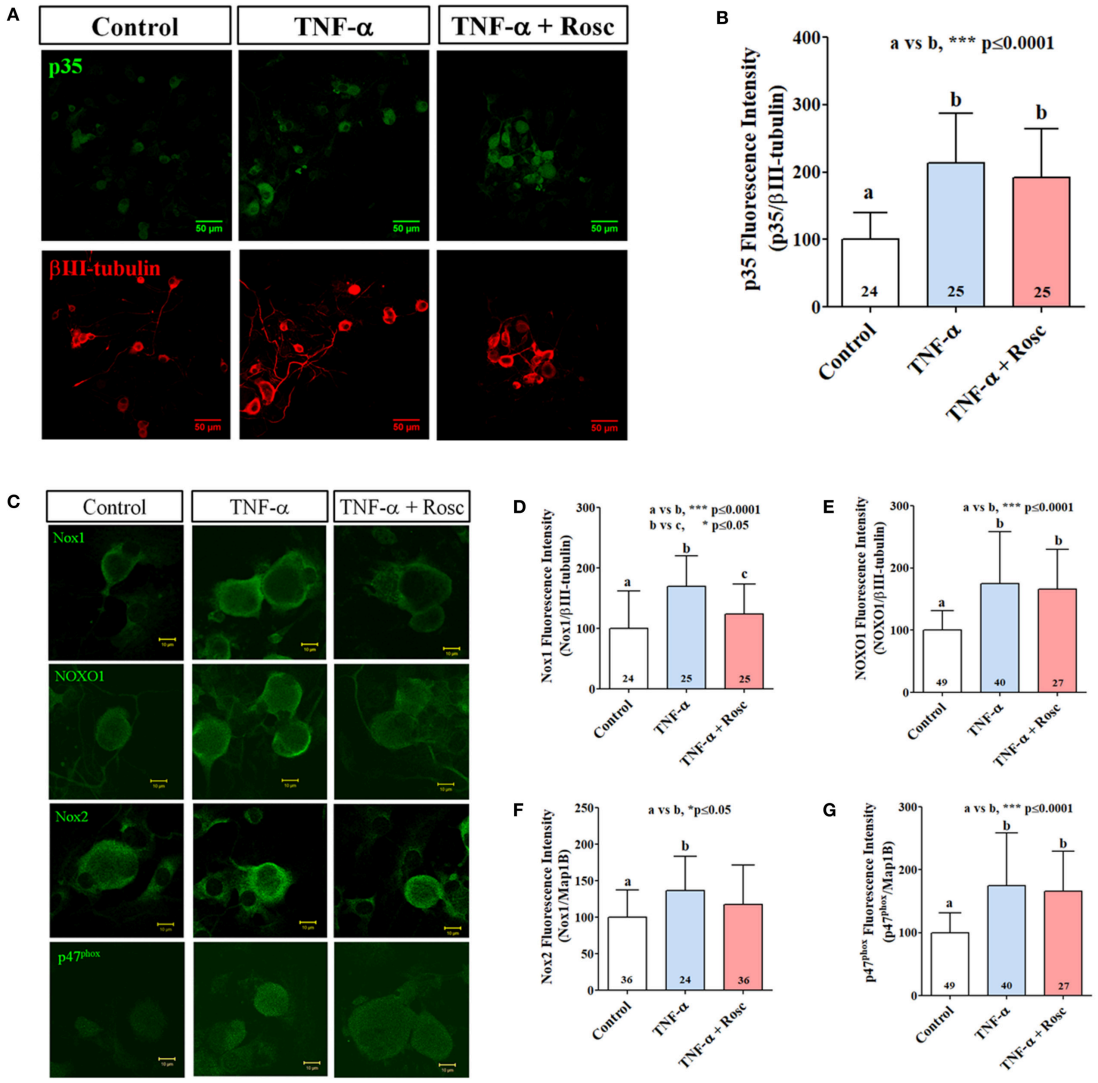
TNF-α treatment increases p35, Nox1 and Nox2 expression in primary culture of mouse TG neurons. **(A)** Representative immunolocalization of p35 (green) and βIII-tubulin (red) from TG neurons treated with TNF-α alone or with roscovitine. Scale bars are 50 μm. **(B)** Quantification of p35 fluorescence intensity of images in **(A)**. **(C)** Representative images of Nox1, NOXO1, Nox2, and p47^phox^ endogenous expression in TG neurons treated with TNF-α alone or with roscovitine. Scale bars are 10 μm. **(D–G)** Quantification of Nox1 **(D)**, NOXO1 **(E)**, Nox2 **(F)**, and p47^phox^ **(G)** fluorescence intensity in TG neurons of images in **(C)**. The bar graphs represent mean ± *SD*. Statistical differences correspond to a one-way ANOVA with a Bonferroni's multiple comparison test. Number of analyzed neurons is inside each corresponding bar.

Because TNF-α increased ROS production in nociceptive neurons, presumably by NOX/NADPH oxidase increased activation, we analyzed the catalytic and cytoplasmic subunits of NOX1 and NOX2 complexes in primary TG cultures treated with TNF-α by immunofluorescence. First, we confirmed that TNF-α increased p35 immunodetection in primary culture of TG neurons (Figures 5A,B) similarly as previously reported (Rozas et al., 2016). We also found that TNF-α treatment increased immunodetection of Nox1, NOXO1, Nox2, and p47^phox^ in TG neurons. Interestingly, roscovitine treatment significantly blocked TNF-α effect in the immunodetection of Nox1 in cultured TG neurons. On the other hand, immunodetection of NOXO1, Nox2, and p47^phox^ was not affected by roscovitine treatment (Figures 5C–G).

### TNF-α increased NOXO1 translocation to plasma membrane in primary culture of mouse TG neurons

**Figure 6:**
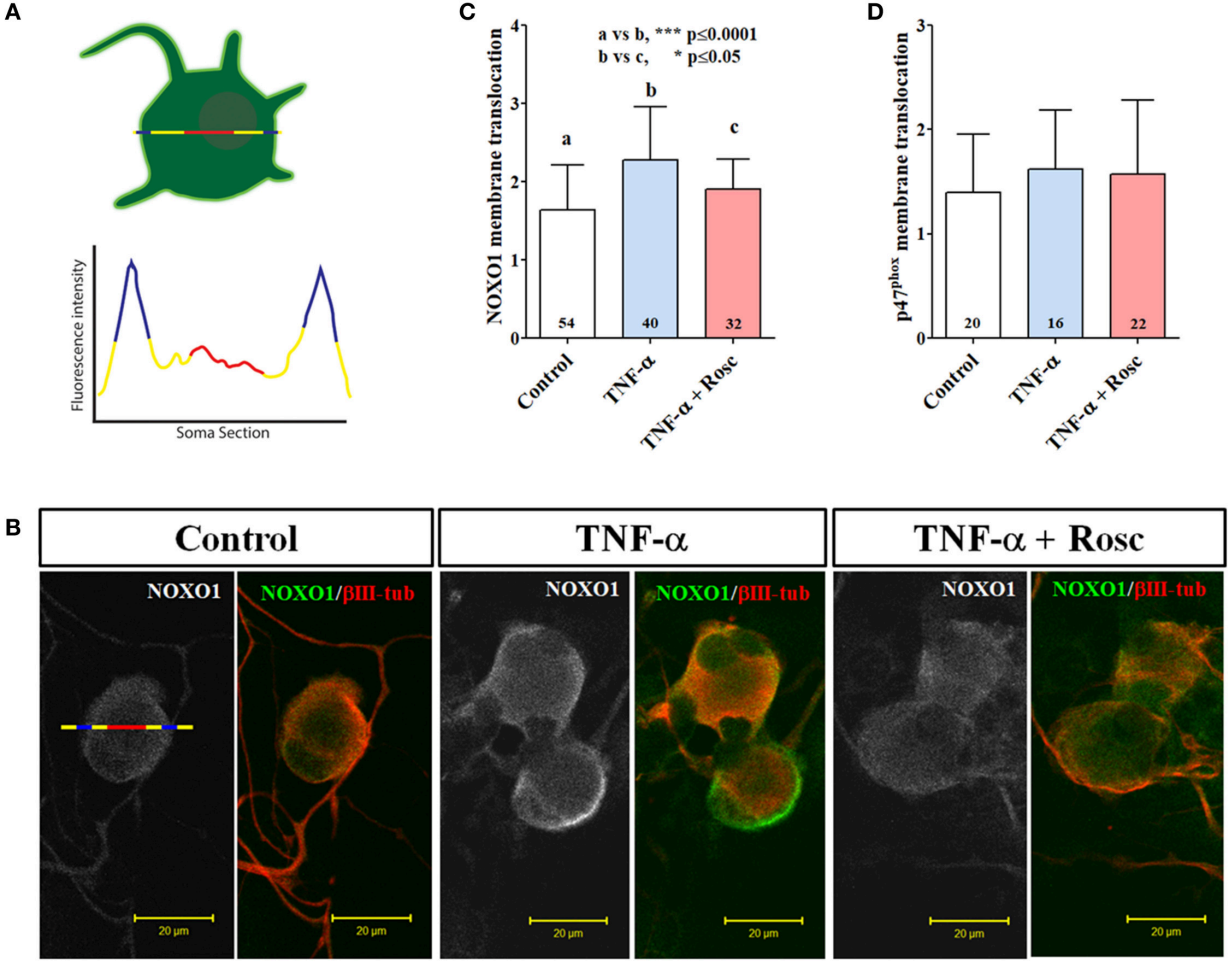
TNF-α increases NOXO1 translocation to plasma membrane in primary culture of mouse TG neurons. **(A)** Upper panel: Descriptive sketch for measurement of plasma membrane translocation of cytosolic NOX subunits. Line represents the measurement of plasma membrane protein translocation indicating a peripheral area (blue section) followed for a centric area (red section). Lower panel: Descriptive plot profile showing measurement of cytosolic NOX along 2D neuron surface. **(B)** Representative immunofluorescences of NOXO1 (white/green) and βIII-tubulin (red) from TG neurons treated with TNF-α (50 ng/ml) alone or with roscovitine (20 mM) during 24 h. Scale bars are 20 μm. **(C,D)** Measurement of NOXO1 **(B)** and p47^phox^ **(D)** translocation to plasma membrane in 2DIV primary culture of TG neurons treated with TNF-α alone or with roscovitine. Plasma membrane translocation was measured from fluorescence intensity into plasma membrane (peripheral area) of TG neurons, normalized against fluorescence intensity in the cytosolic compartment (centric area). The bar graphs represent mean ± *SD*. Statistical differences correspond to a one-way ANOVA with a Bonferroni's multiple comparison test. Number of analyzed neurons is inside each corresponding bar.

Our results showed a significantly increased NOX-dependent ROS production in nociceptive neurons directed by TNF-α signaling and in part by Cdk5 activation. However, an important aspect in NOX complex activation is the recruitment of cytoplasmic subunits to plasma membrane (Debbabi et al., 2013). Therefore, we analyzed the plasma membrane translocation (Figures 6A,B) of cytoplasmic subunits NOXO1 and p47^phox^ after TNF-α treatment by using confocal images (Figure 5C). These analyses revealed that NOXO1 translocation to plasma membrane was enhanced upon TNF-α treatment (Figure 6C). In contrast, we did not find a clear change in its distribution toward peripheral regions for p47^phox^ (Figure 6D). Interestingly, roscovitine treatment reverted TNF-α effect on NOXO1 plasma membrane translocation (Figures 6B,C), which suggests a role of Cdk5 in the recruitment of NOXO1 to peripheral compartments in primary cultured nociceptive neurons.

## Discussion

**Figure 7:**
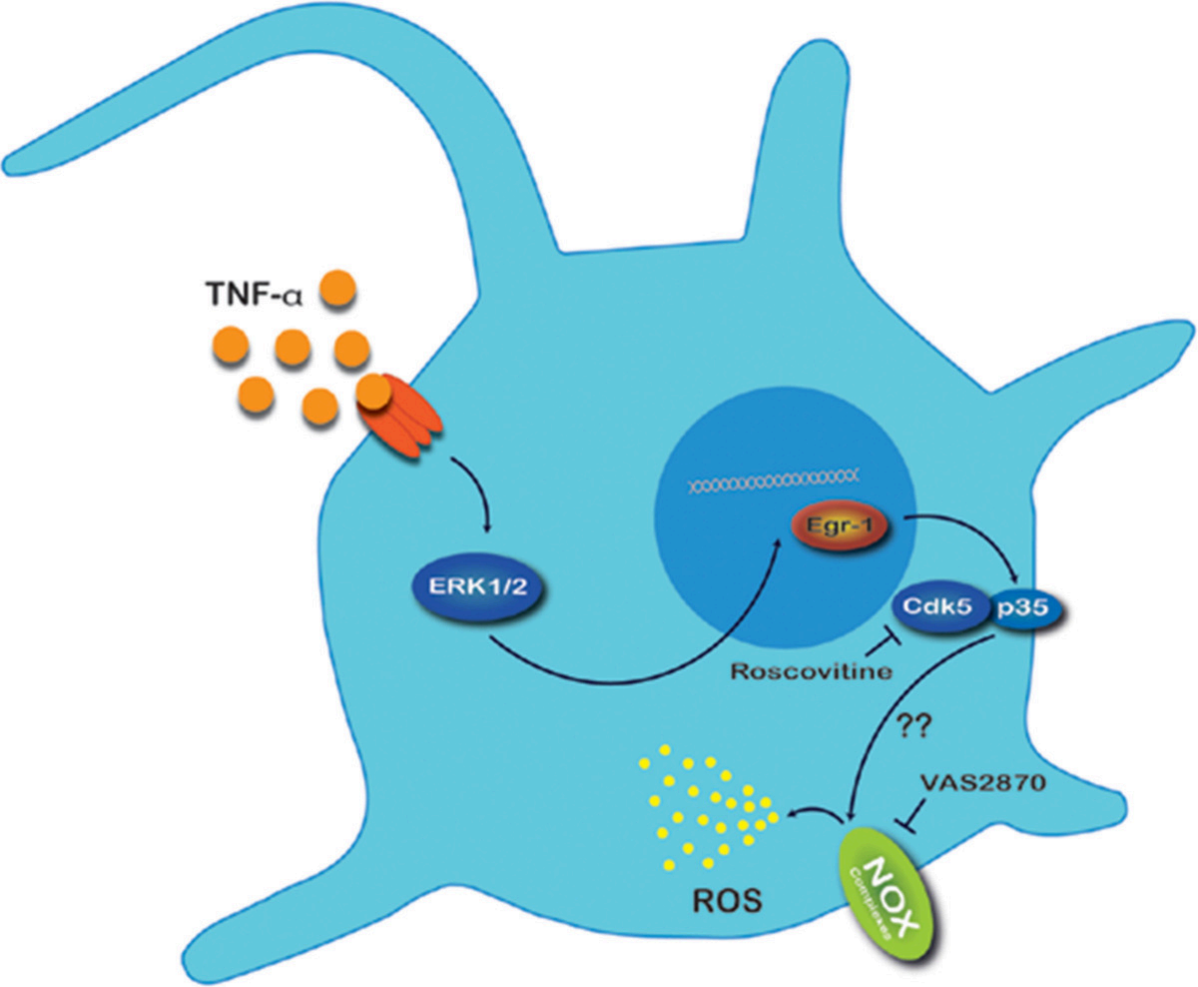
Proposed mechanism for ROS production in nociceptive neurons exposed to TNF-α. Under an inflammatory state, primary sensory neurons are exposed to a pro-inflammatory cytokines soup. TNF-α binds to TNF-α receptor and triggers the MAPK/ERK1/2 pathway. ERK1/2 translocate to nucleus promoting the expression of the transcription factor Early growth response protein 1 (Egr1) which consecutively binds to the p35 promoter causing an increase of p35 protein expression. Activation of Cdk5 by p35 leads to phosphorylation of diverse substrates located in nociceptive neurons; we suggest that this event favors NOX complex association and consequently increases ROS production in these neurons. As ROS represent signaling molecules in pain hypersensitivity, we speculate that this redox imbalance could modify functions of other proteins involved in signal transmission of nociceptive neurons.

In the present work we established for the first time an association between Cdk5 activation and ROS production directed by NOX1 and NOX2 complexes suggesting an important role during inflammatory pain (Figure 7). First, in HEK293 cells transfected with p35, a heterologous expression system, the activation of Cdk5 promotes hydrogen peroxide production that was reverted by pharmacological inhibition of NOX complex or by inhibition of Cdk5 activity. Second, in primary culture of mouse nociceptive neurons, an endogenous expression system, TNF-α treatment increased ROS production and this effect was reverted by inhibition of Cdk5 or NOX complex. Third, TNF-α treatment increased the expression of catalytic (Nox1 and Nox2) and cytosolic (NOXO1 and p47^phox^) members of NOX1 and NOX2 complexes in TG neurons. Moreover, TNF-α treatment induced NOXO1 plasma membrane translocation and roscovitine blocked this effect. Altogether these results demonstrate that Cdk5 contributes to ROS production mediated by NOX1 and NOX2 activation and suggest its involvement during inflammatory pain.

Few years ago, our group established the participation of Cdk5 in pain signaling (Pareek et al., 2006; Utreras et al., 2009a) principally after initiation of an inflammatory response (Utreras et al., 2009b, 2011, 2012; Rozas et al., 2016). In particular, Cdk5 phosphorylates many substrates important in pain such as TRPV1 (Pareek et al., 2007; Jendryke et al., 2016; Rozas et al., 2016), P2X2aR (Coddou et al., 2017), KIF13B (Xing et al., 2012), delta opioid receptors (Xie et al., 2009), among others. Cdk5 phosphorylates TRPV1 in Thr407 decreasing its activation threshold (Jendryke et al., 2016; Rozas et al., 2016). Similarly, Cdk5 phosphorylates purinergic receptor P2X2aR in Thr372 slowing desensitization of the channel (Coddou et al., 2017). Interestingly, both TRPV1 and P2X2aR are ion channels related with hypersensitization during inflammatory pain (Linley et al., 2010). On the other hand, ROS molecules play an important role during inflammatory and neuropathic pain (Kallenborn-Gerhardt et al., 2013), however, the link between ROS production and Cdk5 activation has not yet been addressed until now.

Our results showed that direct activation of Cdk5 by p35 overexpression significantly increased intracellular ROS production in HEK293 cells. Since VAS2870 treatment significantly decreases this redox imbalance, NOX complex raises as a good candidate of ROS source in HEK293 cells. Several members of NOX1 and NOX2 complexes were immunodetected in HEK293 cells, which supports the participation of these enzymes in ROS production mediated by Cdk5 activation. In addition, we detected a NOX-dependent basal production of hydrogen peroxide by HyPer sensor in these cells, because NOX inhibition with VAS2870 significantly decreased ROS production in untransfected cells. Since VAS2870 treatment did not revert Cdk5-mediated ROS levels totally, it could be explained by the activation of other sources of ROS induced by p35 overexpression. In addition, ROS production was not completely abolished in HEK293 cells transfected with p35 and treated with roscovitine, probably because Cdk5 activation by p35 overexpression overcomes the inhibition capacity of roscovitine and a fraction of Cdk5 remained active. Interestingly, this effect was not observed in primary culture of TG neurons where p35 levels were considerably smaller as compared with our heterologous expression system. Moreover, higher concentration of roscovitine could generate a toxic effect on the cells or favor non-specific inhibition of other biological pathways (Bach et al., 2005; Li et al., 2008).

We reported earlier that overexpression of TNF-α in nociceptive neurons increases p35 expression and Cdk5 activity, with a subsequent TRPV1 phosphorylation and an increment in pain signaling (Rozas et al., 2016). Here, we evaluated whether increased Cdk5 activity mediated by TNF-α enhances ROS production in nociceptive neurons. Our results showed that TNF-α treatment significantly increased ROS production in DRG and TG neurons approximately in 50% as compared with control neurons. Similar to HEK293 cells, both roscovitine and VAS2870 treatment reverted ROS production induced by TNF-α, which supports a molecular link between Cdk5 and NOX complexes function, and establishes a potential contribution of Cdk5 activation in the redox balance of nociceptive neurons. Most important, participation of NOX complexes in pain signaling has been already reported (Ibi et al., 2008; Kim et al., 2010; Im et al., 2012b; Kallenborn-Gerhardt et al., 2012; Lim et al., 2013; Kallenborn-Gerhardt et al., 2014). However, only few reports are linked to pro-inflammatory activation (Ibi et al., 2008; Lim et al., 2013). On the other hand, there is evidence that pro-inflammatory cytokines like TNF-α modulate NOX-dependent ROS production in different types of cells (Chen et al., 2008; Lin et al., 2015a; Blaser et al., 2016) including neurons (Barth et al., 2009, 2012; Kuhn, 2014). In addition, interleukin 1β and interleukin 6 have been involved in ROS production mediated by NOX enzymes in different cellular models (So et al., 2007; Kim et al., 2010; Pang et al., 2012; Kuhn, 2014). Our data suggests that increased ROS production mediated by TNF-α treatment could be explained by a higher expression of NOX1 and NOX2 members in nociceptive neurons similarly to previous reports (Kim et al., 2007, 2010; Blaser et al., 2016). However, NOX4 represents an important source of ROS in DRG neurons contributing to pain hypersensitization (Im et al., 2012a; Kallenborn-Gerhardt et al., 2012; Ding et al., 2017), moreover regulating TRPV1 activity (Lin et al., 2015b; Ding et al., 2016). Therefore, this study cannot exclude the possibility that NOX4 participates in Cdk5-mediated ROS production.

NOX cytosolic members NOXO1 and p47^phox^ can undergo post-translational modification by phosphorylation dependent of PKC (Fontayne et al., 2002; Debbabi et al., 2013) inducing NOX activation and ROS production. Interestingly, we found a minimal consensus sequence (Ser/Thr-Pro) for Cdk5 phosphorylation in mouse p47^phox^ protein (Ser215 and Thr356) and a full consensus sequence for Cdk5 (Ser/Thr(Pro)X(Lis/His/Arg) (Bórquez et al., 2013) in NOXO1 protein (Ser3). Therefore, we postulate that Cdk5 upon activation could phosphorylate cytosolic subunits of NOX1 and NOX2, promoting activation and ROS production, although further experiments are needed to demonstrate such a novel regulation.

Considering this scenario, we think that Cdk5-mediated ROS production in nociceptive neurons could contribute to enhancing pain signaling by an additional mechanism. Interestingly, several receptors expressed on the surface of TG and DRG neurons are susceptible to activity modulation by cysteine oxidation, such as TRPV1, transient receptor potential ankyrin 1 (TRPA1), N-methyl-d-aspartate (NMDA) receptors, and T-type Ca^2+^ channels, among others (Gamper and Ooi, 2015). However additional experiments are needed to demonstrate the real impact of Cdk5-mediated ROS production in the nociceptive circuitry including the central nervous system. In summary, taken together our results suggest that Cdk5 activation may be implicated in the ROS production by NOX1 and NOX2 complexes during inflammatory pain and this relationship could address additional roles to Cdk5 in pain signaling.

## Author contributions

RS, CG-B, and EU designed the project. RS, PL, FF, NP, and EU performed the experiments. RS, PL, FF, CG-B, and EU analyzed the data. RS, CG-B, and EU wrote the manuscript. EU supervised the experiments and finalized the manuscript.

### Conflict of interest statement

The authors declare that the research was conducted in the absence of any commercial or financial relationships that could be construed as a potential conflict of interest.
